# A Breathable, Low-Cost, and Highly Stretchable Medical-Textile Strain Sensor for Human Motion and Plant Growth Monitoring

**DOI:** 10.3390/s26010044

**Published:** 2025-12-20

**Authors:** Shilei Liu, Xin Wang, Xingze Chen, Zhixiang He, Linpeng Liu, Xiaohu Jiang

**Affiliations:** 1 Hunan Agricultural Forestal and Industrial Prospective Design Institute, Co., Ltd., Changsha 410007, China; liushilei0610@foxmail.com; 2 State Key Laboratory of Precision Manufacturing for Extreme Service Performance, College of Mechanical and Electrical Engineering, Central South University, Changsha 410083, China; 3 College of Mechanical and Electrical Engineering, Hunan Agricultural University, Changsha 410128, China

**Keywords:** strain sensor, highly stretchable, low cost, plant growth monitoring

## Abstract

Flexible strain sensors capable of conformal integration with living organisms are essential for advanced wearable electronics, human–machine interaction, and plant health. However, many existing sensors require complex fabrication or rely on non-breathable elastomer substrates that interfere with the physiological microenvironment of skin or plant tissues. Here, we present a low-cost, breathable, and highly stretchable strain sensor constructed from biomedical materials, in which a double-layer medical elastic bandage serves as the porous substrate and an intermediate conductive medical elastic tape impregnated with carbon nanotubes (CNTs) ink acts as the sensing layer. Owing to the hierarchical textile porosity and the deformable CNTs percolation network, the sensor achieves a wide strain range of 100%, a gauge factor of up to 2.72, and excellent nonlinear second-order fitting (R^2^ = 0.997). The bandage substrate provides superior air permeability, allowing long-term attachment without obstructing moisture and gas exchange, which is particularly important for maintaining skin comfort and preventing disturbances to plant epidermal physiology. Demonstrations in human joint-motion monitoring and real-time plant growth detection highlight the device’s versatility and biological compatibility. This work offers a simple, low-cost yet effective alternative to sophisticated strain sensors designed for human monitoring and plant growth monitoring, providing a scalable route toward multifunctional wearable sensing platforms.

## 1. Introduction

Wearable strain sensors are important components in the growing field of flexible electronics, providing real-time information about mechanical deformation during human movement [1,2,3,4,5] and the growth of plant stems or leaves [6,7,8,9,10,11]. Their ability to conform to irregular three-dimensional surfaces and convert strain into electrical signals has facilitated progress in areas such as personalized healthcare [12,13,14], prosthetics [15,16,17], sports monitoring [18,19,20], soft robotics [21,22], and precision agriculture [23,24,25,26,27,28]. Over the past decade, advances in functional materials and device structures have enabled sensors with improved mechanical flexibility, wider detection ranges, and more stable signal output [29]. Nanomaterials including graphene, CNTs, MXenes, and conductive polymers have been widely explored due to their good electrical conductivity, flexibility, and compatibility with soft biological surfaces [30,31,32,33]. For example, laser-induced graphene sensors show good sensitivity and durability in monitoring skin signals [34], and highly stretchable polymer- or fiber-based devices attached to leaves, stems, or fruits allow non-destructive and continuous tracking of plant growth, contributing to applications in phenotyping and environmental sensing [27,35,36].

Although much progress has been made, the broader use of wearable strain sensors in practical human and plant applications is still limited by several key issues. One major challenge is the widespread reliance on dense and non-breathable elastomer substrates such as PDMS, Ecoflex, or SEBS [37,38,39]. These materials are easy to process and mechanically tunable, but their impermeability interferes with heat transfer, moisture evaporation, and gas exchange at the sensor–tissue interface. This often leads to skin irritation, sweat accumulation, and thermal discomfort during long-term wear, together with instability of sensing signals. The situation can be more serious for plants, where non-breathable substrates disturb local microenvironments, affect transpiration and humidity distribution, and mechanically restrict natural growth, thereby influencing both plant physiology and sensing accuracy. These problems make it difficult to achieve long-term and reliable monitoring, which is essential for health tracking, rehabilitation, and continuous agricultural observation.

Another challenge arises from the fabrication complexity of many high-performance strain sensors. Methods such as photolithography, direct ink writing, multilayer transfer, laser processing, microcrack engineering, or hybrid micro/nano-structuring often require specialized equipment, controlled environments, or multiple assembly steps [29,40]. These techniques can produce high-quality devices but also increase cost and reduce scalability, limiting their use in practical or large-scale applications. Devices that rely on engineered microstructures or multilayer composites may also experience interfacial delamination, long-term signal drift, or reduced durability under repeated deformation, especially in outdoor or humid environments.

These limitations highlight the need for strain sensors that combine good stretchability, biocompatibility, breathability, environmental stability, and simple, low-cost manufacturing. Textile-based substrates have recently gained attention due to their intrinsic breathability [41,42]. However, many textile-integrated sensors still depend on additional coatings, complex patterning steps, or polymer encapsulation, which can reduce air permeability or complicate fabrication. A practical solution should balance several features including breathable and compliant substrates to maintain the natural microenvironment of skin or plant tissues, conductive networks that remain electrically stable under large deformations, and fabrication processes that avoid complicated or expensive steps.

To meet these requirements, this work introduces a sensor architecture based entirely on common medical-grade materials. The device uses a double-layer medical elastic bandage as a porous and compliant substrate, combined with an elastic medical tape coated with CNT ink to form a deformable conductive network. The woven bandage structure provides good air and moisture transport and helps maintain a stable biological interface, while the CNT coating offers reliable electromechanical response across a wide strain range. This materials-based design achieves a practical balance between sensitivity, durability, and conformability without requiring complex fabrication. The sensor reaches a maximum strain of 100%, a gauge factor of 2.72, and a second-order fitting accuracy of R^2^ = 0.997. Compared with traditional elastomer-based sensors, the porous bandage provides better comfort and reduces interference with human skin or plant tissues during long-term attachment. The CNT-based conductive layer ensures stable signal output and mechanical robustness under repeated or dynamic deformation. Demonstrations involving human joint monitoring and plant growth tracking confirm the effectiveness of this approach and its potential for use in various biological environments. Overall, this strategy offers a simple, low-cost, and scalable path for developing breathable, biocompatible, and versatile strain sensors suitable for continuous monitoring in both human and plant systems.

## 2. Materials and Methods

### 2.1. Materials

Elastic self-adhesive bandages (Yiwu Bence Daily Necessities Co., Ltd., Yiwu, China) were purchased from a local pharmacy. Anhydrous ethanol was obtained from Macklin. Carbon nanotubes (CNTs) were supplied by Suzhou Tanfeng Graphene Technology Co., Ltd., Suzhou, China.

### 2.2. Fabrication of the Sensor

**Figure 1:**
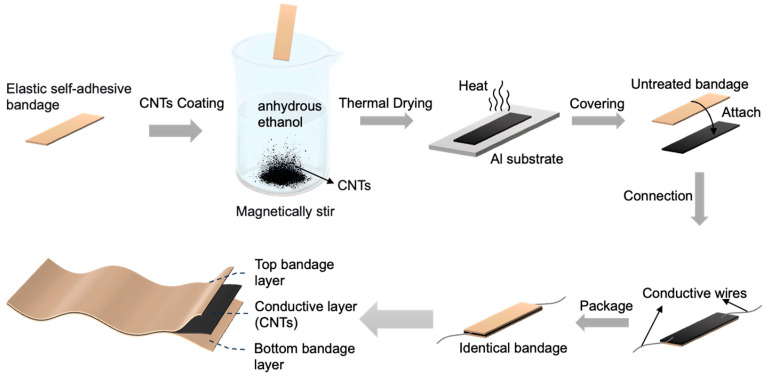
Preparation method of the breathable, highly sensitive, flexible strain sensor.

The elastic self-adhesive bandage was cut into strips measuring 1 cm × 6 cm, immersed in anhydrous ethanol, and ultrasonically cleaned (Shenzhen Fuyang Technology Group Co., Ltd., Shenzhen, China) for 10 min to remove surface impurities. The bandages were subsequently dried on a heating plate (Dongguan Bangyuan Electronics Co., Ltd., Dongguan, China) at 100 °C for 10 min to ensure complete elimination of residual solvents. To prepare the conductive ink, 0.5 g of CNTs (Suzhou Tanfeng Graphene Technology Co., Ltd., Suzhou, China) was dispersed in 50 g of anhydrous ethanol, followed by ultrasonic treatment for 10 min. The pretreated bandages were then introduced into the dispersion, which was magnetically stirred (Joan Lab Equipment (Zhejiang) Co., Ltd., Huzhou, China) at 500 rpm for 2 h to facilitate uniform CNT adsorption. After stirring, the coated bandages were removed and dried at 100 °C for 20 min until completely solvent-free. Finally, a clean bandage of identical dimensions was laminated onto the CNT-coated layer for encapsulation, and electrical leads were attached to yield the flexible strain sensor. The fabrication process of the flexible strain sensor is illustrated in Figure 1.

### 2.3. Performance Test and Characterization of Sensors

To systematically evaluate the mechanical performance of the sensor device, tensile tests were conducted on the sensing layer using a compact desktop universal testing machine (ZQ-950, Dongguan Zhicai Precision Instrument Co., Ltd., Dongguan, China). The surface morphology of the sensing layer under both unstretched and stretched states was characterized using a 3D digital optical microscope (VHX5000, Keyence Corporation, Osaka, Japan). A stepping-motor-driven system (Suzhou Mingkun Technology Co., Ltd., Suzhou, China) was employed to measure resistance variations across different strain intervals, enabling a comprehensive assessment of the sensor’s strain-response characteristics over the full detection range. Additionally, a digital multimeter (Keithley DMM6500, Tektronix Co., Ltd., Shanghai, China) was used to monitor resistance changes during various human motions and to record the resistance responses of plants under complex real-world conditions.

## 3. Results and Discussion

### 3.1. Electromechanical Response of the Sensor

**Figure 2:**
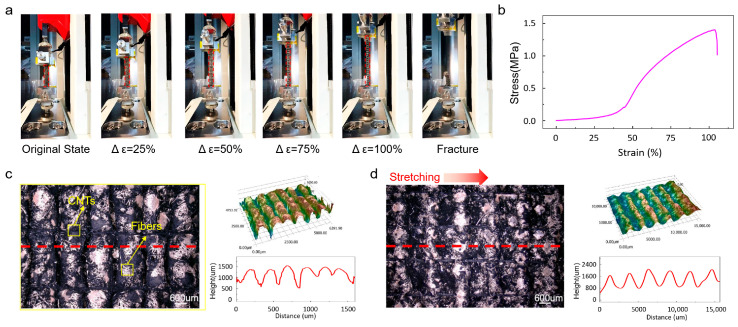
Mechanical performance and mechanism analysis of the sensor device. (**a**) Photograph of the sensing layer being stretched on a universal testing machine. (**b**) Stress–strain curve of the sensor device. (**c**) Morphology of the sensing layer in the unstretched state observed under a 3D digital microscope, along with the schematic illustration of the sensing mechanism. The geometric contour map is derived from the position of the red dotted line on the planar topography map. (**d**) Comparison of the sensing layer morphology under unstretched and stretched states observed using a 3D digital microscope. The geometric contour map is derived from the position of the red dotted line on the planar topography map.

To evaluate the mechanical properties of the CNT-modified cotton-fiber sensing layer, uniaxial tensile tests were conducted using a universal testing machine. Figure 2a shows the evolution of the sensing layer under different stretching states, including 0%, 25%, 50%, 75%, and 100% strain, and the final fracture condition. The corresponding stress–strain curve is presented in Figure 2b. The results indicate that the CNT-treated cotton layer exhibits an elongation at break exceeding 100%, together with a maximum tensile strength of approximately 1.2 MPa, demonstrating good mechanical robustness.

To further explore the relationship between its structural characteristics and the sensing mechanism, the surface morphology of the sensing layer in the undeformed state was examined using a 3D optical microscope (Figure 2c). The CNTs are clearly observed to coat the cotton fibers and form an interconnected three-dimensional conductive network. Upon lateral stretching, the inherent wrinkles within the cotton fibers gradually unfold and extend, leading to changes in the number of contact points and the effective length of conductive pathways. These structural changes result in a corresponding increase in electrical resistance. Figure 2d compares the morphology before and after stretching, showing an obvious lateral expansion of the cotton-fiber layer. This observation agrees well with the proposed mechanism in which resistance changes originate primarily from the reconstruction of the conductive network during fiber deformation.

**Figure 3:**
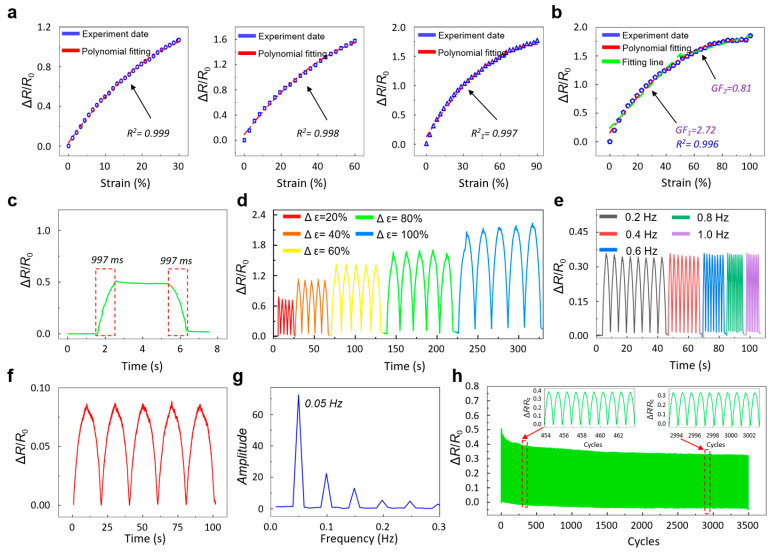
Strain-sensing performance of the sensor. (**a**) Relative resistance variation in the sensor at strain levels of 30%, 60%, and 90%. (**b**) Resistance variation in the sensor over the full strain range up to 100%. (**c**) Response and recovery time of the sensor under 15% strain. (**d**) Relative resistance change in the sensor during cyclic stretching at different strain levels. (**e**) Cyclic response of the sensor under 10% tensile strain at frequencies ranging from 0.3 to 2.4 Hz. (**f**,**g**) Resistance variation in the sensor under an external force of 0.25 Hz and the corresponding electrical signal obtained via fast Fourier transform. (**h**) Long-term stability test of the sensor under 10% strain for 3500 loading cycles. The insets show enlarged views of resistance variations during the 454–464th and 2993–3000th cycles.

To evaluate the electromechanical response of the sensor across its full strain range, systematic tests were conducted under different deformation levels. As shown in Figure 3a, polynomial fitting was performed for three representative strain intervals of 30%, 60%, and 90%. The corresponding coefficients of determination (R^2^ = 0.999, 0.998, and 0.997) indicate that the sensor maintains consistent resistance–strain behavior throughout these strain regions. When tested over the entire strain range up to 100%, the device continues to exhibit a high degree of fitting accuracy (R^2^ = 0.996), suggesting stable and reliable output across large deformations. To further examine the variation in sensitivity, a segmented linear fitting was conducted with 50% strain as the boundary (Figure 3b). The gauge factor is calculated to be 2.77 in the 0–50% range and 0.81 in the 50–100% range, indicating that the sensor exhibits different but predictable response characteristics in the low- and high-strain regimes.

Beyond static evaluation, the dynamic response characteristics of the sensor were also studied. Under a strain of 15%, the response time was measured to be 997 ms (Figure 3c). To assess repeatability, cyclic stretching tests were performed at strain levels of 20%, 40%, 60%, 80%, and 100%, with five cycles for each level. As shown in Figure 3d, the resistance curves for repeated loading and unloading overlap closely within each strain condition, demonstrating good repeatability and signal stability.

Frequency-dependent behavior was evaluated under a fixed strain of 10% across frequencies from 0.2 Hz to 1.0 Hz (Figure 3e). The resistance variations remain consistent across different frequencies, suggesting that the sensor maintains stable signal fidelity within this bandwidth. To further investigate its low-frequency detection capability, tests were performed at 5% strain and 0.05 Hz. As shown in Figure 3f, the sensor continues to generate clear and stable waveforms at this low frequency. A Fourier transform of the signal (Figure 3g) confirms that the sensor can reliably detect strain excitations as low as 0.05 Hz. Long-term mechanical durability was examined through 3500 consecutive loading–unloading cycles at a strain amplitude of 10% (Figure 3h). Throughout the test, the peak resistance variation remains stable with minimal fluctuation, and no noticeable signal degradation or drift was observed. These results indicate that the sensor maintains stable electromechanical performance under prolonged cyclic loading, meeting the reliability requirements for practical long-term applications.

**Figure 4:**
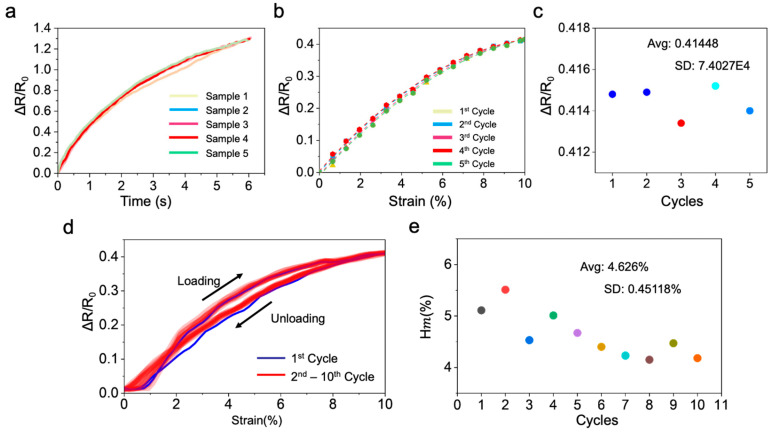
(**a**) Consistency of the fabricated sensors. (**b**) △R/R_0_-strain curves of the sensors under 5 strain-loading cycles (10% strain). (**c**) Scatter plot of the peak relative resistance change in the sensor under repeated 10% strain loading cycles. Each point corresponds to a cycled peak under 10% strain in (**b**). (**d**) △R/R_0_-strain curves of the sensors under 10 cycles (10% strain). (**e**) Hysteresis error of the sensor. Each point corresponds to a hysteresis error under a loading-unloading cycle in (**d**).

In addition, the consistency, repeatability, and hysteresis error of the fabricated sensors are tested, respectively. Five sensors were stretched from 0 strain to 40% strain and recording their respective relative resistance change rates, respectively, as shown in Figure 4a. It can be seen that the curves of each sensor have a high degree of overlap, demonstrating good consistency. And a sensor was repeatedly stretched from 0 to 10% strain for five cycles under identical testing conditions, and the corresponding relationships between the relative resistance change and strain were recorded, as shown in Figure 4b. The maximum values obtained from these five curves were further extracted and summarized in Figure 4c. The calculated mean value is 0.414446, with a standard deviation (SD) of 0.00074027, indicating excellent repeatability of the sensor. For the sensor’s hysteresis error, we conducted 10 cycles of tensile tests on the sensor, with a strain of 10% for each stretch. The relationship results between the relative resistance change rate and strain obtained are shown in Figure 4d. After further statistical analysis, the average hysteresis error of the sensor obtained was 4.626%, while the SD was 0.45118%.

### 3.2. Applications of the Sensor in Human Motion Monitoring

**Figure 5:**
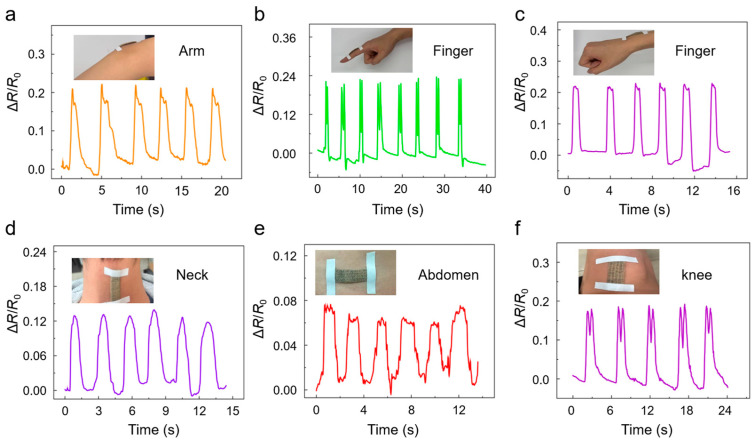
Sensor applied for monitoring various human physiological motions. (**a**–**f**) Resistance variation during elbow joint movement, neck flexion and extension, wrist flexion–extension, knee flexion–extension, abdominal expansion during breathing, and finger-joint bending, respectively.

The flexible strain sensor demonstrates robust capability for capturing a wide spectrum of human motion signals, ranging from large-amplitude joint bending to subtle deformations. As shown in Figure 5a–c, when the sensor is attached to highly mobile joints such as the arm (Figure 5a), the neck (Figure 5b), and the wrist (Figure 5c), it produces distinct and repeatable relative resistance variations that correspond closely to the flexion and extension of each joint. The periodic and stable waveforms confirm the sensor’s excellent strain sensitivity and mechanical compliance for monitoring large dynamic deformations.

In addition to large-amplitude movements, the sensor can also detect small motions. When placed on regions with small or slow movements, such as the knee and the abdomen in Figure 5d,e, it generates clear resistance fluctuations that accurately reflect subtle knee bending (Figure 5d) and abdominal expansion during breathing (Figure 5e). The sensor also maintains high signal fidelity on curved and irregular surfaces, as illustrated by the stable responses recorded during finger motion monitoring in Figure 5f. The results presented in Figure 5a–f demonstrate the high sensitivity, strong motion correlation, and excellent adaptability of the sensor across a wide range of human-motion scenarios, highlighting its promise for wearable health monitoring and soft human–machine interfaces.

A series of experiments was conducted to assess the sensor’s performance under static deformation, continuous biological growth, and dynamic environmental perturbations. This set of tests was designed to examine the sensor’s stability, linear response behavior, and applicability in plant monitoring scenarios.

**Figure 6:**
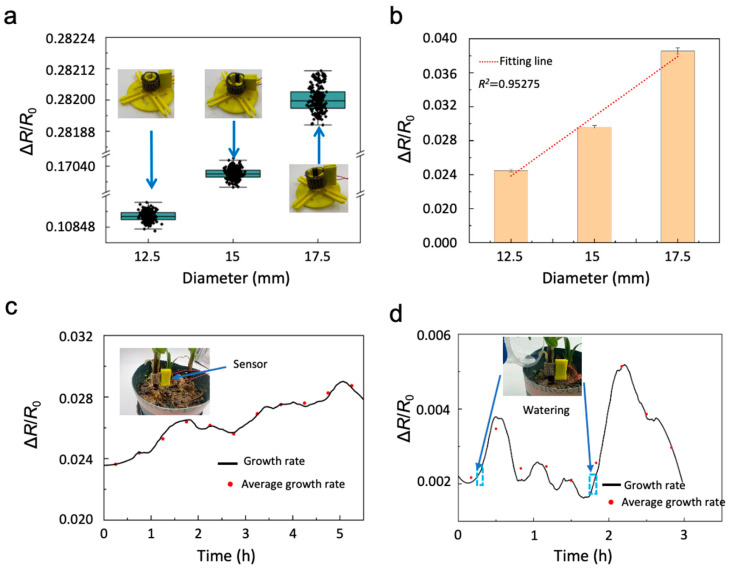
Sensor performance in practical application scenarios. (**a**,**b**) Relative resistance variation when the sensor is attached to cylindrical objects of different diameters. (**c**) Real-time tracking of plant (Lily) stem response over 6 h with the strain sensor attached. (**d**) Monitoring the plant stem response for 3 h after water supplementation while the sensor remains attached.

Static evaluation was performed using a diameter-adjustable platform with fixed deformation states of 12.5 mm, 15 mm, and 17.5 mm. For each diameter, continuous measurements were collected for two hours to simulate long-term operation under fixed strain conditions. As shown in Figure 6a, the distributions under the three deformation states remain tightly constrained, with fluctuation ranges below 0.00005. Such highly compact data clusters confirm that the sensor maintains remarkable signal stability in static environments, effectively minimizing the influence of noise and intrinsic drift. To examine the degree of linearity, the mean values at each diameter were analyzed through regression, yielding a coefficient of determination of 0.95274 (Figure 6b). This result suggests that the sensor exhibits a consistent response trend across the tested strain range.

### 3.3. Applications of the Sensor in Plant Growth Monitoring

Following the static evaluation, the sensor was applied in a natural, non-intervention plant-growth monitoring scenario to assess its capability for long-term, nondestructive biological sensing. For stretchable strain sensors, measuring human joint motion and monitoring plant stem growth are fundamentally based on the same sensing mechanism. The difference lies mainly in the deformation profile: joint motion involves rapid, cyclic stretching and relaxation, whereas plant stem growth results in slow, unidirectional bending or radial expansion. In both cases, the essential principle is that mechanical stretching of the sensor substrate induces resistance changes in the embedded conductive sensing network, and these resistance variations follow the same basic principles described by Ohm’s law. Consequently, the sensor must be capable of detecting the larger strains generated during human joint motion while also resolving the slow, small-strain changes associated with plant stem thickening. Continuous measurements were acquired over a six-hour period. As shown in Figure 6c, the resistance variation displays a characteristic pattern that rises rapidly to a peak before gradually declining. The general trend of the resistance curve is consistent with the expected slow variation associated with plant stem expansion. To further examine stability, averaged values were taken every thirty minutes, and these points closely follow the overall curve. This indicates that the sensor is capable of capturing small, progressive changes during plant growth within the tested timescale.

To evaluate the response to dynamic biological changes, a watering experiment was conducted during a three-hour monitoring session. Water was applied at the twentieth and one hundredth minute. As illustrated in Figure 6d, each watering event resulted in a noticeable increase in the resistance variation, followed by a gradual return toward the baseline. Averaged values collected every fifteen minutes show a trend similar to the raw data. These observations indicate that the sensor can register transient changes in plant stem behavior associated with water uptake within the measured period.

Overall, the tests confirm that the sensor provides stable output under static deformation and responds consistently across different deformation states. In plant monitoring scenarios, it is capable of detecting gradual growth-associated changes as well as short-term physiological variations after watering. These results suggest that the sensor is suitable for applications involving long-term growth observation and environmental-response monitoring in plants.

## 4. Conclusions

In summary, we have developed a breathable, low-cost, and highly stretchable strain sensor based entirely on medical elastic textiles and a graphene/CNT hybrid conductive ink. The synergy between the porous woven bandage substrate and the deformable G/CNT sensing layer enables high mechanical compliance, robust conductivity, and excellent sensing performance, including a working strain of 100%, a gauge factor of 2.72, and strong second-order nonlinear fitting (R^2^ = 0.997). Unlike conventional elastomer-based sensors, the bandage-structured substrate allows continuous moisture and gas exchange, minimizing physiological disturbance and enabling long-term attachment to both human skin and plant surfaces. The sensor successfully captures a range of human motions and plant growth behaviors, demonstrating its versatility across biological systems. Compared to sophisticated flexible graphene sensors for human monitoring and ultrastretchable devices designed for plant growth tracking, our approach offers a simple, accessible, and scalable alternative that retains competitive performance while dramatically reducing fabrication complexity. The findings presented here suggest that medical-textile-based architectures may represent a promising direction for the next generation of multifunctional wearable strain sensors, enabling widespread deployment in healthcare, environmental monitoring, and intelligent agriculture.

## Data Availability

The original contributions presented in the study are included in the article; further inquiries can be directed to the corresponding author.
